# Estimating the potential contribution of stroke treatments and preventative policies to reduce the stroke and ischemic heart disease mortality in Turkey up to 2032: a modelling study

**DOI:** 10.1186/s12889-015-2655-8

**Published:** 2016-01-19

**Authors:** Duygu Islek, Kaan Sozmen, Belgin Unal, Maria Guzman-Castillo, Ilonca Vaartjes, Julia Critchley, Simon Capewell, Martin O’Flaherty

**Affiliations:** 1 Department of Public Health, Dokuz Eylul University, Faculty of Medicine, İzmir, Turkey; 2 Department of Public Health, Faculty of Medicine, Katip Celebi University, İzmir, Turkey; 3 Institute of Psychology, Health & Society, University of Liverpool, Liverpool, UK; 4 Deparment of Public Health and Policy, Institute of Psychology, Health and Society, University of Liverpool, Liverpool, UK

**Keywords:** Stroke, Cardiovascular Diseases, Death, Decision Modeling, Prevention

## Abstract

**Background:**

Stroke and Ischemic Heart Diseases (IHD) are the main cause of premature deaths globally, including Turkey. There is substantial potential to reduce stroke and IHD mortality burden; particularly by improving diet and health behaviours at the population level. Our aim is to estimate and compare the potential impact of ischemic stroke treatment vs population level policies on ischemic stroke and IHD deaths in Turkey if achieved like other developed countries up to 2022 and 2032.

**Methods:**

We developed a Markov model for the Turkish population aged >35 years. The model follows the population over a time horizon of 10 and 20 years. We modelled seven policy scenarios: a baseline scenario, three ischemic stroke treatment improvement scenarios and three population level policy intervention scenarios (based on target reductions in dietary salt, transfat and unsaturated fat intake, smoking prevalence and increases in fruit and vegetable consumption). Parameter uncertainty was explored by including probabilistic sensitivity analysis.

**Results:**

In the baseline scenario, we forecast that approximately 655,180 ischemic stroke and IHD deaths (306,500 in men; 348,600 in women) may occur in the age group of 35–94 between 2012 and 2022 in Turkey. Feasible interventions in population level policies might prevent approximately 108,000 (62,580–326,700) fewer stroke and IHD deaths. This could result in approximately a 17 % reduction in total stroke and IHD deaths in 2022. Approximately 32 %, 29 %, 11 % and 6 % of that figure could be attributed to a decreased consumption of transfat, dietary salt, saturated fats and fall in smoking prevalence and 22 % could be attributed to increased fruit and vegetable consumption. Feasible improvements in ischemic stroke treatment could prevent approximately 9 % fewer ischemic stroke and IHD deaths by 2022.

**Conclusions:**

Our modeling study suggests that effective and evidence-based food policies at the population level could massively contribute to reduction in ischemic stroke and IHD mortality in a decade and deliver bigger gains compared to healthcare based interventions for primary and secondary prevention.

**Electronic supplementary material:**

The online version of this article (doi:10.1186/s12889-015-2655-8) contains supplementary material, which is available to authorized users.

## Background

Ischemic stroke and ischemic heart disease (IHD) continue to cause most cardiovascular and circulatory deaths globally, which mostly contribute to the increasing number of premature deaths, [1]. Stroke accounts for a substantial proportion of these deaths worldwide, with being the main cause of 10 % of the deaths and 4 % of the DALYs (Disability adjusted life years) in 2010, [2]. While some high-income countries show dramatic decreases in stroke incidence in the past four decades, in many low and middle income countries stroke incidence and number of stroke related deaths and DALYs are still increasing, [2, 3].

In common with other middle income countries, stroke is still one of the most common cause of deaths in Turkey. In 2013, IHD and stroke remained to be the top two causes of years of life lost (YLLs) as they were in National Burden of Study in 2000, [4, 5].

Stroke and IHD deaths are eminently preventable and acute and secondary treatments contribute to reduce mortality, however, prevention from stroke could contribute to this reduction more. Consumption of a diet with reduced sodium, increased portions of fruits and vegetables, with smoking cessation and with control of diabetes mellitus in combination with treatment of hypertension shown to prevent stroke incidence and deaths, [6]. These kind of effective interventions may require policy initiatives to support the individuals to change health behaviours or to effectively medicate them, [7]. Thus, a better understanding of the effectiveness of policy or other interventions has been an urgent need to inform ischemic stroke and IHD prevention strategies.

We, therefore, aimed to model and compare the future impact of different policy scenarios targeting to improve ischemic stroke treatment and to improve nutrition at the population level with policy interventions including smoking cessation on both ischemic stroke and IHD deaths for the next two decades.

## Methods

**Fig. 1:**
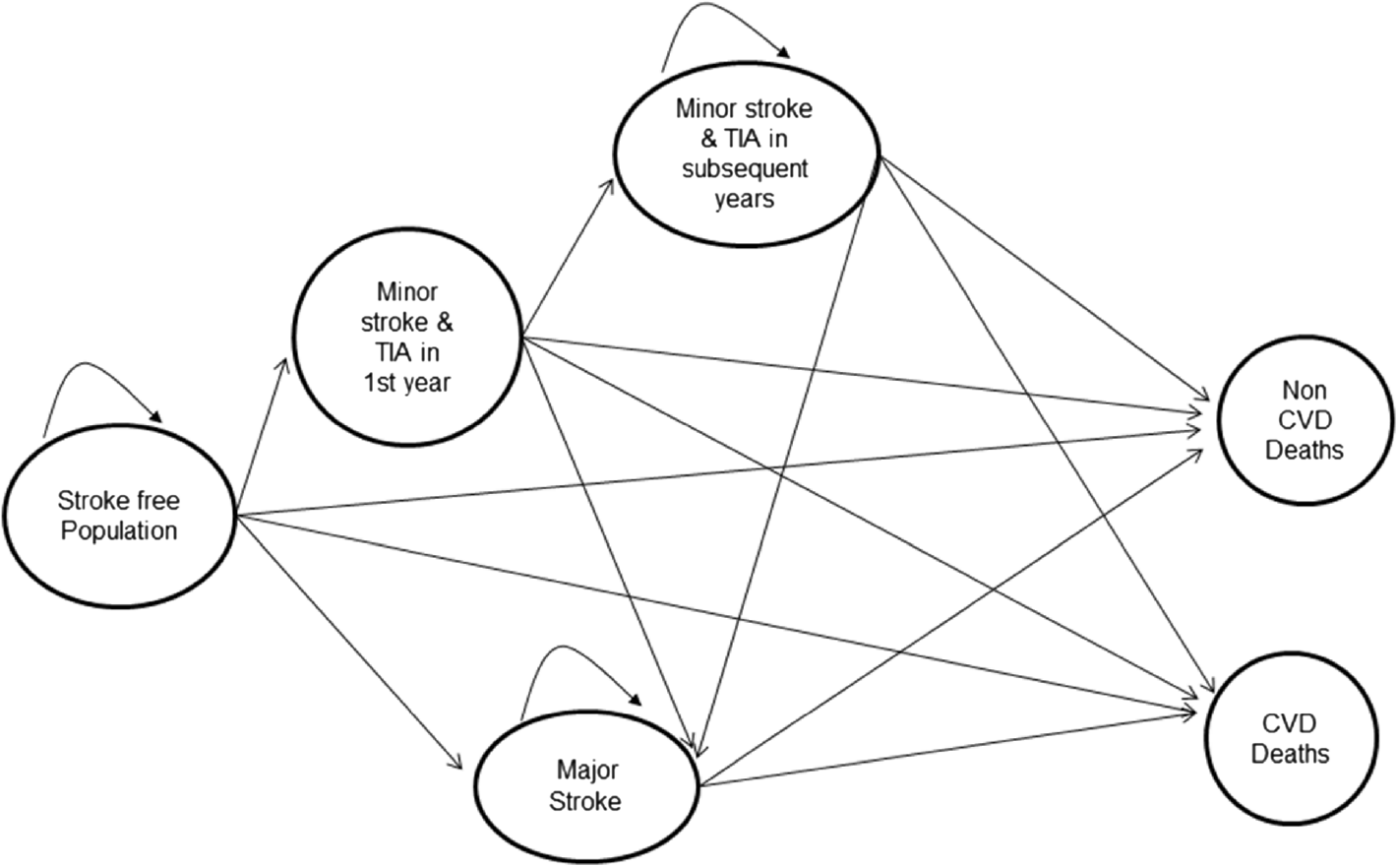
The relations of health states in ischemic stroke model. TIA: Transient Ischemic Attack. CVD: Cardiovascular Disease

The Ischemic Stroke Model is a Microsoft Excel cell-based Markov model, for Turkish population of 35 years and over, consisting finite number of health states reflecting the natural history of ischemic stroke. Markov Model provides estimates for future burden of CVD deaths and it can provide information for policy makers by comparing the outcomes from different intervention scenarios targeting specific risk factors and treatments [8, 9]. The model starts in 2012 and runs for 10 and 20 years. We assumed that a close cohort of individuals is free of ischemic stroke at the start of the simulation and every year the individuals can move from one health state to another. The possible pathways between heath states are shown in Fig. 1.

The main model inputs are population data, incidence and case-fatality rates of ischemic stroke. The main outcome is the total number of ischemic stroke and IHD deaths that will occur over a 10 and 20 years time horizon.

Ethical approval was not required for this model based study as we made secondary analysis of previously published, unidentified and publicly available data.

### Data sources

#### Data for population and prevalence of stroke

We obtained the population data for year 2012 from Turkish Statistical Institute (TurkStat), [10]. We obtained prevalence of ischemic stroke from a nationwide study conducted by Ministry of Health in 2012, [11]. Using the population and ischemic stroke prevalence data, we calculated the number of ischemic stroke patients in total population in 2012. By this way we calculated the number of cohort population that is free of stroke at the start year 2012.

#### Data for incidence of stroke

We modelled the incidence of stroke in Turkey for this stroke-free population. Since there was no available data for incidence of stroke in Turkey, we estimated stroke incidence using DISMOD II software [12]. DISMOD II calculates the incidence by using data on age and gender-specific stroke prevalence, total population, total mortality rates and stroke remission rate (assumed to be zero). DISMOD II software also allows us to input incidence from a similar population in order to make a better estimation. We therefore used incidence from an Iranian stroke cohort study [13] and finally found an estimate for Turkey.

#### Data for mortality and case-fatality rates

We obtained number of deaths for age groups 35 to 94 from TurkStat for the year 2012, [10]. The model also required total Cardiovascular Diseases (CVD) deaths data with the ICD 10 codes from I00 to I99 which we also obtained from TurkStat database for year 2012. The case fatality rates of ischemic stroke (both minor and major) were calculated by analyzing the hospital based Aegean Stroke Registry database with Kaplan-Meier Method. Aegean Stroke Registry was used with the collaboration of researchers of Egean University Hospital [14, 15]. All the input data were specified for age and sex.

### Intervention scenarios

We modelled seven scenarios: a baseline scenario, three medical treatment scenarios and three population level prevention scenarios.

For the baseline scenario we assumed no change will happen during 10 and 20 years in the present uptake rates of medical therapies or population level uptakes of nutrients and population smoking prevalence.

For optimal scenarios to prevent ischemic stroke and heart diseases, either with medical treatment or preventetive policies, we first simulated the achievements of more developed countries as the best scenario target [16–22]. After we were clear about the optimal scenario standards; we simulated a modest improvement in treatments and interventions; based on expert opinions (cardiologist and neurologist) as a standard local evidence was not available. We then calculated a feasible scenario, half way between the optimal and conservative ones.

### Medical treatment scenarios

- an optimal scenario; We simulated an increase in the uptake levels that are already reached in more developed countries, using as an an exemplar country, the Netherlands [16, 17].
- a conservative scenario; We assumed a modest 10 % relative increase in the current uptake levels.
- a feasible scenario; We simulated a more realistic change, halfway between the current situation and the optimistic scenario.

The simulated scenarios for the uptake rates of medical treatments can be seen in Table 1.

**Table 1:** Scenarios for the uptake rates of medical therapies in Turkish population

	Baseline scenario (uptake rates in 2012)	Conservative scenario targets	Feasible scenario targets	Optimal scenario targets
Acute stroke treatment				
Thrombolysis	0,01	0,011	0,05	0,13
Aspirin	0,50	0,55	0,70	0,85
Stroke unit	0,00	0,01	0,40	0,85
Secondary prevention				
Aspirin	0,26	0,29	0,50	0,81
Statin	0,48	0,53	0,70	0,80^a^
Warfarin	0,14	0,20	0,40	0,50^a^
BP control	0,35	0,39	0,50	0,62
Smoking cessation	0,30	0,33	0,50	0,60^a^
Primary prevention				
BP control	0,07	0,08	0,10	0,12^a^
HbA1c control	0,49	0,54	0,70	0,80^a^
Smoking cessation	0,04	0,07	0,10	0,13
Warfarin	0,30	0,33	0,50	0,78

^a^Assuming 10 % relative increase in scenarios

### Population level food policy scenarios

In optimal scenario we assumed improvements already achieved in exemplar countries. A salt intake reduction of 30 % (as in Finland and Japan), [18, 19], which we simulated as a reduction of 5 g/day from the current salt consumption in Turkey, (14.8 g/day), [23], a 15 % decrease in the prevalence of smoking (Australia and California), [20, 21]. and an absolute decrease of 5 % in energy from saturated fats (Finland), [22]. In the conservative scenario we assumed a small decrease (1 g/day) in salt intake, and 0.5 % decrease in energy from transfat and 1 % from saturated fats (replacing it with energy from mono—and polyunsaturates), a modest 5 % decrease in the prevalence of smoking. Finally, in the feasible scenario we simulated reductions between the conservative and optimistic ones (Table 2).

**Table 2:** Scenarios for population level policy interventions

Scenarios	Conservative	Feasible	Optimal
Reduction in Salt Intake (g/day)	1	3	5
Reduction in Trans-Fat Intake (%)	0.5	1	2
Reduction in Saturated Fat (%) (replaced by polyunsaturates)	1	3	5
Increase in Fruit And Vegetable Intake (portion/day)	1	2	3
Reduction in Smoking Prevalence (%)	5	10	15

### Modelling policy effectiveness and its impact in mortality

The model applies the relative risk reduction (RRR) quantified in previous randomised controlled trials and meta-analyses based on overseas studies. The model calculates the reductions in ischemic stroke and heart disease deaths by multiplying the transition probabilities with policy effectiveness. Table 3 shows the relative risk reductions for ischemic stroke and IHD deaths applied for each intervention scenario.

**Table 3:** Relative risk reductions for CVD deaths from previous studies for intervention scenarios

Intervention	Relative risk reduction (RRR)	Description
Thrombolysis Treatment	11 % (95 % CI: 5–16)	RRR for stroke death or dependency if applied within 4.5 h [48]
Aspirin Treatment	2,6 % (95 % CI: 0.4–4)	RRR for stroke death or dependency if treatment is 160–300 mg once daily, started within 48 h of onset [49]
Stroke Unit	6,1 % (95 % CI:0,0009–11)	RRR for stroke death or dependency [50]
Aspirin Treatment for secondary prevention	3 % (95 % CI: 6–19)	RRR for vascular events (stroke or IHD death) if treatment is at any dose above 30 mg daily [51]
Statin Treatment	12 % (95 % CI: (-1)–21)	RRR for recurrent stroke if LDL reduces by 1 mmol/L [52]
Warfarin Treatment for secondary prevention	61 % (95 % CI: 37–75)	RRR for recurrent stroke or systemic embolism among stroke patients with Transient Ischemic Attack or minor stroke due to atrial fibrillation when treated with anticougulation [53]
BP Control for secondary prevention	34 % (95 % CI: 21–44)	RRR for stroke based on BP reduction of 4–25 mmHG systolic or 3–13 Hg diastolic [54]
Smoking Cessation	48 % (95 % CI: 29–57)	RRR for stroke death or dependency [16]
BP Control for primary prevention	46 % (95 % CI: 35–55)	RRR based on BP reduction 5 mmHg. This reduces the risk of stroke by an estimated 34 % and ischemic heart disease by 21 % from any pre-treatment level [55]
HbA1C Control	7 % (95 % CI: 4–19)	RRR for stroke based on 0,9 % HbA1C reduction [56]
Warfarin Treatment for primary prevention	64 % (95 % CI: 49–74)	RRR for stroke based on a meta-analysis with twenty-nine trials, adjusted-dose warfarin reduced stroke by 64 % [57]
Salt Reduction	17 % (95 % CI:6–43)	RRR for stroke by 5gr change in daily salt intake [58]
Transfat Reduction	12 % (95 % CI:5.5–18.5)	RRR by replacing 1 % of energy from trans-fat with unsaturated fats for coronary heart disease [59] We assumed half effect for stroke
Saturated Fat Reduction	13 % (95 % CI: 1–6)	RRR by replacing 5 % of energy from saturated fat with Polyunsaturated fats (PUFAs) for coronary heart disease [60] We assumed half effect for stroke
Fruit and Vegetables Intake	4 % (95 % CI:3–8)	RRR for stroke by change in 1 unit of fruit and vegetables [61]
Smoking Prevalence Reduction	1.9 % (95 % CI:1.5–2.3)	RRR by change in 1 % prevalence of smoking [62]

Ischemic heart disease deaths were classified under the ICD 10 codes I20–I25. An example of the calculation method used for calculating the deaths prevented or postponed (DPPs) with a population level policy scenario is available in Additional file 1, section 1.5. We modelled all the intervention scenarios to calculate the total number CVD deaths (ischemic stroke and IHD deaths) that could be prevented or postponed under each specific scenario, compared to the baseline scenario.

### Sensitivity analysis

We implemented sensitivity analysis using the Excel add-in Ersatz software which allows Monte Carlo simulation, [24]. This allowed us to calculate 95 % uncertainty intervals (95 % UI) for prevented or postponed deaths, based on 1000 draws from specified probabilistic distributions for the model input variables.

Further details on methods and data sources are provided in Additional file 1.

## Results

In the baseline scenario, we forecast that approximately 655,180 ischemic stroke and IHD deaths (306,500 in men; 348,600 in women) may occur in the age group of 35–94 between 2012 and 2022 and 1,150,000 ischemic stroke and IHD deaths (544,720 in men; 650,230 in women) between 2012 and 2032 in Turkey.

**Fig. 2:**
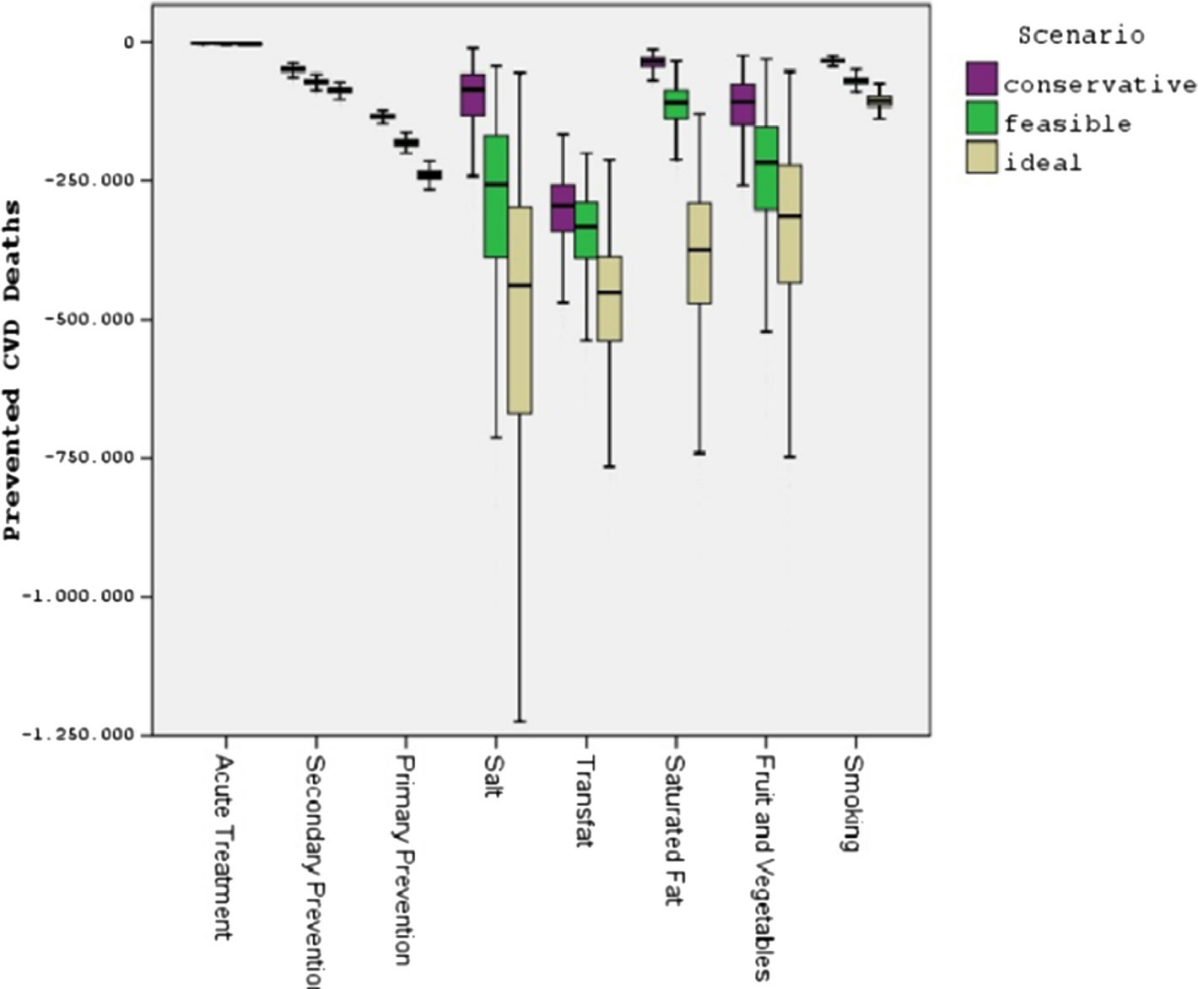
Reduction in number of deaths by achievement of improvements in Turkey up to 2022. CVD: Cardiovascular Disease

Figure 2 shows the estimated number of the ischemic stroke and IHD deaths that could be prevented or postponed by achievement of conservative, feasible or optimal scenarios of medical treatment and population level policy improvements in Turkey for year 2022.

### Optimal scenario

Optimal improvements in acute treatment, secondary and primary prevention of stroke could result in approximately 89,810 fewer ischemic stroke and IHD deaths (74,725–97,432) in 10 years. This would represent a 13 % reduction in forecasted ischemic stroke and IHD deaths up to year 2022 and continuing up to 2032 in Turkey.

A daily reduction of 5 g/day in salt intake could result in approximately 51,975 fewer ischemic stroke and IHD deaths (range: 6,257–283,596). A reduction of 2 % in total energy from trans fats might generate approximately 47,528 fewer deaths (range:22,581–125,960) while a reduction of 5 % in total energy from saturated fats could result in approximately 39,563 fewer deaths (range:12,924–126,526). Additional 3 portions of fruit and vegetables daily consumption could result in approximately 35,893 fewer deaths (range:7,480–183,191) and a decrease of 15 % in the smoking prevelance of total population in 10 years might result 10,739 (range:7,551–15,118) fewer deaths up to year 2022 and continuing up to 2032 in Turkey.

### Conservative scenario

Slight improvements in acute treatment, secondary and primary prevention of ischemic stroke; assuming 10 % relative increase in the present uptake levels of medical therapies could result in approximately 49,885 fewer ischemic stroke and IHD deaths (45,757–61,714) in 10 years. This would represent a 7 % reduction from the total forecasted stroke and IHD deaths up to 2022 and a 9 % reduction up to 2032 in Turkey.

Reducing the total energy from trans fats by 0.5 % and from saturated fat by 1 %, reducing salt consumption by 1 g per day, and increasing fruit and vegetable intake by 1 portion per day and 5 % decrease in smoking prevalence could result in approximately 58,962 fewer ischemic stroke and IHD deaths (minimum: 30,786 maximum: 119,990) in 10 years and 103,993 fewer deaths (54,288–212,091) in 20 years. This would represent an 11 % reduction in the total forecasted stroke and IHD deaths up to year 2022 and continuing up to 2032 in Turkey.

### Feasible scenario

Table 4 shows the estimated reductions in number of ischemic stroke and IHD deaths with achievement of a feasible scenario in acute, secondary and primary levels of ischemic stroke treatment.

**Table 4:** Reduction in number of deaths with achievement of feasible treatment interventions in Turkey

	2022				2032			
	Men		Women		Men		Women	
	N (min-max)	%	N (min-max)	%	N (min-max)	%	N (min-max)	%
Acute Stroke treatment	290 (97–2310)	1.1	975 (385–2700)	2.0	1030 (345–890)	2.0	2160 (725–6425)	2.4
Secondary Prevention	7310 (5920–15930)	28.2	19520 (15945–35495)	39.0	27620 (22430–57335)	53.0	51280 (42165–91435)	58.0
Primary prevention	18260 (15590–20505)	70.6	29620 (25855–33095)	59.1	23080 (20570–26250)	45.0	34940 (31115–40090)	39.6
Total	25860 (22640–32415)	100.0	50115 (38900–64635)	100.0	51730 (47995–80115)	100.0	88380 (76960–143020)	100.0

Achievement of improvements in treatment of ischemic stroke could result in approximately a 9 % reduction in total forecasted ischemic stroke and IHD deaths up to year 2022.

Table 5 shows the estimated reduction in number of ischemic stroke and IHD deaths by achievement of a feasible scenario (simulating reductions in dietary salt by 3 g/day trans-fat by 1 % of daily energy intake saturated fat by 3 % of daily energy intake increasing fruit and vegetable intake by 2 portions/day and 10 % decrease in smoking prevalence in the population). These population level policy interventions could result in approximately a 17 % reduction in total forecasted ischemic stroke and IHD deaths up to year 2022 and continuing up to 2032.

**Table 5:** Reduction in number of deaths with achievement of feasible population level policy interventions in Turkey

Policy Options	2022				2032			
	Men		Women		Men		Women	
	N (min-max)	%	N (min-max)	%	N (min-max)	%	N (min-max)	%
Reduction in Salt Intake	16670 (1300–105490)	29.0	14220 (2340–80580)	28.0	27390 (2130–177990)	30.0	23245 (3820–133140)	28.0
Reduction in Transfat Intake	18680 (12995–35170)	32.0	16090 (5730–37800)	32.0	24790 (14920–53655)	28.0	26320 (9360–62030)	32.0
Reduction in Saturated Fat Intake	6340 (2240–19970)	11.0	5395 (1905–17050)	11.0	10380 (3665–32850)	11.0	8800 (3110–27885)	11.0
Increasing Fruit & Vegetables	13050 (2825–44075)	22.0	10780 (1880–43140)	22.0	21415 (4630–72990)	24.0	18120 (3150–73255)	22.0
Reduction in Smoking Prevalence	3640 (2535–5075)	6.0	3490 (2580–5009)	7.0	6475 (4510–9040)	7.0	6100 (4510–8760)	7.0
Total	58370 (25000–114695)	100.0	49980 (32590–124140)	100.0	90450 (44720–205545)	100.0	82580 (57780–218130)	100.0

## Dıscussıon

This modelling study estimates the future impact of possible population level policy interventions and ischemic stroke treatment scenarios on ischemic stroke and IHD deaths in Turkey. Approximately 655000 ischemic stroke and IHD deaths may occur between 2012 and 2022 in the base scenario. The model estimates suggest that even with conservative changes in food policy approximately 60000 deaths can be prevented or postponed in 10 years time. Primary prevention and food policies especially feasible reductions in transfat and salt made the highest contribution to this health gain whereas acute and chronic care for ischemic stroke with contributing additional benefits will be unsufficient to significantly impact on future burden of stroke in Turkey.

Many of the interventions to prevent deaths from IHD and ischemic stroke constitute medical treatments and the “high-risk approach”. However there could be massive health gains by prevention of IHD and ischemc stroke deaths if population level policies were successfully implemented in Turkey.

Our study suggests that thousands of deaths could be prevented with transfat reduction at population level. This could be achieved by public campaigns food labelling and legislative policies [25]. There are exampler countries who imposed legislation on food manufacturers to limit their population’s intake of trans-fats. For example in 2003 Denmark set an upper limit for artificial trans fat levels in foods effectively banning partially hydrogenated oils. In the following years New York and Switzerland introduced legislation to limit trans fat, [26, 27]. In both Denmark and New York legislation has effectively eliminated industrial transfats; for example in New York restaurants the prevalence of use of industrial transfats has declined from 50 % to less than 2 % [28, 29]. Similarly in the UK while transfat accounted for 1.1 % of food energy in 2000 this reduced to 0.8 % of food energy in 2010 with implementation of successful legislative strategies [30]. There are also successful examples of policy interventions on industrial food labelling. In 2006 introduction of mandatory trans fat labelling in US prompted reformulation and many products are now labelled as free from transfats [25]. Although there have been discussions on effectiveness of transfat reduction strategies for example suggesting that legislative strategies have been more successful than labelling or education [28, 29] the findings of a review with data from developed countries show that the policies introduced to decrease transfat levels in food supply have been effective regardless of the intervention employed [31]. However there is sparse evidence for low and middle income countries on effectiveness of these policy strategies responses and health outcomes. Furthermore important inequalities exist with high consumption of trans fats in disadvantaged groups thus benefits in deprived communities might be larger [32]. In Turkey Turkish Ministry of Health has recently established strategic plans on transfat labeling and reformulation for the year 2017, [33]. Our findings show that the health gains of these actions will be massive in Turkey as a middle income country if effectively implemented.

Reduction of salt in populations has been a prior concern for prevention of cardiovascular diseases including IHD and ischemic stroke. The WHO Member States agreed on a voluntary global target for a 30 % relative reduction in mean population intake of salt with the aim of achieving a target of less than 5 g per day by 2025. Many countries including Japan UK Finland and Portugal have reduced population-wide salt intake in the recent years through a combination of regulations on the salt content in processed foods labeling of processed and prepared foods public education and engagement with the food industry [34]. In the UK for example salt reduction strategy started in 2004 and the adult daily salt intake decreased from 9.5 g/day to 8.6 g/day by May 2008 [30]. This was a 10 % population-reduction in salt achieved over 4 years without reduction in sales of the products and without consumer complaints about taste [35]. In Finland and Japan 6 g/day reduction was achieved with specific legislation [34]. Likewise in most developed countries a reduction in salt intake could be achieved by reduction in the amount of salt added to food by the industry. However in some countries public health campaigns might be needed to raise awareness of population where most of the salt comes from salt added during cooking [34]. In Turkey for example where 30 % of daily salt consumption comes from bread a 3 gr/day salt reduction (from 18gr/day to 15gr/day) was achieved between 2008 and 2012 both with health campaings and legislation on the amount of salt in bread [23]. Our estimates suggest that with continuation of the achievements in salt reduction at population level even with conservative reductions thousands of IHD and ischemic stroke deaths could be saved in the following two decades.

Our study also suggests that substantial number of IHD and ischemic stroke deaths could be prevented by increasing fruit and vegetable consumption at population level. Although Turkey produces and exports fruit and vegetable the consumption of fruit and vegetable shows a decreasing trend [36] and dietary patterns differ substantially between regions and income groups. Income level and lack of knowledge about healthy nutrition are the main determinants of nutrition patterns in Turkey, [37]. Therefore for low-income groups which suffer the highest burden of IHD and stroke the benefit might even be higher with an increase in daily consumption of fruit and vegetables.

### Comparison with other modeling studies

In two recent modelling studies in the UK and Ireland substantial burden of cardiovascular disease (CVD) deaths might be avoided as a result of changes in average population dietary intakes. They used IMPACT Food Policy Model which had a similar study design to our modelling study [38] [39]. Similar to our estimate of 32 % (the highest) contribution of decrease in trans-fat consumption to the fall in ischemic stroke and IHD deaths O’Keeffee et al estimated the greatest potential impact (28 %) on fall in CVD deaths with a decrease in trans fat consumption followed by impact of saturated fat (22 %) dietary salt (23 %) and increased fruit and vegetable consumption (26 %) in Ireland [38]. Conservative scenarios in food policy could result in a 10 % overall reduction in CVD mortality in Ireland and 8 % reduction in UK [39] very similar to our findings for Turkey using a different modelling approach. Modest reduction in dietary salt could substantially reduce cardiovascular events in other modeling studies similar to our model estimates for Turkish population. Reduction of salt of approximately 2 g/day in Argentina was projected to result in 2.7 % decrease in total stroke mortality in 10 years [40]. In UK reduction of 3 g/day salt would result with 2 % decrease in risk reduction in CVD events with savings worth at least £40 m a year [32].

### Strenghts and limitations of the study

Our study has some strengths. This is the first modelling study on ischemic stroke mortality in Turkey based on available national population-based data. The model is extendable to other settings and populations. We made transparent assumptions with a clear justification. For example as we did not have some of the data that the model required for medical treatment uptake rates and interventions at the population-level we based our uptakes rates from the Netherlands supplemented with expert opinion (listed in Additional file 1: Table S1) Furthermore we used DISMOD II software to estimate stroke incidence that is consistent with prevalence and mortality estimates for the Turkish population. Finally we conducted probabilistic sensitivity analyses to explore the role of uncertainity of model parameters.

Our study also has some limitations. We assumed that risk factor effects were independent which could result in an overestimate of the number of prevented deaths for each policy. Furthermore our model assume that food policies homogeneously effect the entire population and we simulated similar food policy targets across all age groups regardless of morbidity and the socioeconomic variation in Turkey. The model was based on ischemic stroke incidence and number of prevented deaths however the benefit of the people already have non-fatal stroke could have been taken into consideration. Additionally we did not consider the lag between improvements in medical treatments and risk factors changes and corresponding decreases in IHD and ischemic stroke mortality. However there is substantial evidence suggesting that declines in mortality can happen rapidly after individual or population-wide changes in diet or smoking [41].

## Conclusıons

The stroke and cardiovascular disease burden like other low and middle income countries is increasing in Turkey. This public health epidemic requires immediate action. Our analysis suggests that effective and evidence-based food policies at the population level could massively contribute through a 20 % reduction in ischemic stroke and IHD mortality in a decade and deliver bigger gains compared to healthcare based interventions for primary and secondary prevention. Results of previous modelling studies [42, 43] for prevention of cardiovascular diseases have been utilising by the governments and WHO [44, 45]. Turkish Ministry of Health also has policy documents and action plans to reduce the burden of noncommunicable diseases with effective prevention at national level [46, 47]. We particularly used these targets for simulation of scenarios for the next 10 years. Our modelling study can be a useful tool to explore potential benefits of implementing certain strategies for prevention of ischemic stroke and heart diseases in the future. Further studies that include economic analysis of different interventions are needed to compare the cost effectiveness of future scenarios in order to target and implement the best interventions.

## Additional file

Additional file 1:
**Technical Appendix.** (DOC 417 kb)

## References

[1] Mortality GBD (2015). Causes of Death C. Global, regional, and national age-sex specific all-cause and cause-specific mortality for 240 causes of death, 1990-2013: a systematic analysis for the Global Burden of Disease Study 2013. Lancet.

[2] Feigin VL, Forouzanfar MH, Krishnamurthi R, Mensah GA, Connor M, Bennett DA (2014). Global and regional burden of stroke during 1990-2010: findings from the Global Burden of Disease Study 2010. Lancet.

[3] Feigin VL, Lawes CM, Bennett DA, Barker-Collo SL, Parag V (2009). Worldwide stroke incidence and early case fatality reported in 56 population-based studies: a systematic review. Lancet Neurol.

[4] Global Burden of Disease Study C. Global, regional, and national incidence, prevalence, and years lived with disability for 301 acute and chronic diseases and injuries in 188 countries, 1990-2013: a systematic analysis for the Global Burden of Disease Study 2013. Lancet. 2015. doi:10.1016/S0140-6736(15)60692-4 10.1016/S0140-6736(15)60692-4 PMC4561509 26063472

[5] The Ministry of Heath of Turkey, Burden of Disease Study 2004. e-library website. http://ekutuphane.tusak.gov.tr/kitaplar/turkiye_hastalik_yuku_calismasi.pdf. Accessed 5 May 2015.

[6] Lackland DT, Roccella EJ, Deutsch AF, Fornage M, George MG, Howard G (2014). Factors influencing the decline in stroke mortality: a statement from the American Heart Association/American Stroke Association. Stroke.

[7] Modelling the UK burden of cardiovascular disease to 2020 (2008). A Research Report for the Cardio & Vascular Coalition and the British Heart Foundation.

[8] Briggs AH, Claxton K, Sculpher MJ (2006). Decision Modelling for Health Economic Evaluation.

[9] Hunink MGM. Decision Making in Health and Medicine with CD-ROM (2001). Integrating Evidence and Values.

[10] Turkish Statistical Institute: Population Statistics. 2012. http://www.turkstat.gov.tr/UstMenu.do?metod = kategorist Accessed 28 April 2015.

[11] Republic of Turkey. Ministry of Health: Chronic Disease and Risk Factors Survey in Turkey. 2012. http://sbu.saglik.gov.tr/Ekutuphane/kitaplar/khrfai.pdf Accessed: 12 July 2015.

[12] Barendregt JJ, Van Oortmarssen GJ, Vos T, Murray CJ (2003). A generic model for the assessment of disease epidemiology: the computational basis of DisMod II. Population Health Metrics.

[13] Azarpazhooh MR, Etemadi MM, Donnan GA, Mokhber N, Majdi MR, Ghayour-Mobarhan M (2010). Excessive incidence of stroke in Iran: evidence from the Mashhad Stroke Incidence Study (MSIS), a population-based study of stroke in the Middle East. Stroke.

[14] Kumral E, Ozkaya B, Sagduyu A, Sirin H, Vardarli E, Pehlivan M (1998). The Ege Stroke Registry: a hospital-based study in the Aegean region, Izmir, Turkey. Analysis of 2,000 stroke patients. Cerebrovasc Dis.

[15] Altun D, Sozmen K, Damgacı V, Ilhan S, Kumral E, Unal B. Abstract. [Survival in patients who attented to Egean University Hospital with first stroke between years 2008-2009]. Turkish National Public Health Conference.

[16] Hankey GJ (2010). Ischaemic stroke--prevention is better than cure. J R Coll Physicians Edinb.

[17] Ramsay S (2007). Missed opportunities for secondary prevention of cerebrovascular disease in elderly British men from 1999 to 2005: a population-based study. JPublic Health.

[18] Laatikainen T, Pietinen P, Valsta L, Sundvall J, Reinivuo H, Tuomilehto J (2006). Sodium in the Finnish diet: 20-year trends in urinary sodium excretion among the adult population. Eur J Clin Nutr.

[19] Cappuccio FP, Capewell S, Lincoln P, McPherson K (2011). Policy options to reduce population salt intake. BMJ.

[20] Scollo M, Winstanley M (2012). Tobacco in Australia: Facts and issues.

[21] Pierce JP, Messer K, White MM, Kealey S, Cowling DW (2010). Forty years of faster decline in cigarette smoking in California explains current lower lung cancer rates. Cancer Epidemiol Biomarkers Prev.

[22] Pietinen P, Paturi M, Reinivuo H, Tapanainen H, Valsta LM (2010). FINDIET 2007 Survey: energy and nutrient intakes. Public Health Nutr.

[23] Turkish Society of Hypertension and Renal Diseases: Salt Intake Study in Turkey. 2012. www.turkhipertansiyon.org/ppt/SALTurk2.ppt. Accessed 11 Dec 2014.

[24] Ersatz Epigear. Version 1.3. http://www.epigear.com/index_files/ersatz.html Accessed: 14 Feb 2015.

[25] Mozaffarian D, Stampfer MJ (2010). Removing industrial trans fat from foods. BMJ.

[26] British Heart Foundation:Trans fats Policy Statement. https://www.bhf.org.uk/publications/policy-documents/trans-fats-policy-statement---january-2010 Accessed 11 Nov 2014.

[27] Brownell KD, Pomeranz JL (2014). The trans-fat ban--food regulation and long-term health. N Engl J Med.

[28] Leth T, Jensen HG, Mikkelsen AA, Bysted A (2006). The effect of the regulation on trans fatty acid content in Danish food. Atherosclerosis Supplements.

[29] Angell SY, Silver LD, Goldstein GP, Johnson CM, Deitcher DR, Frieden TR (2009). Cholesterol control beyond the clinic: New York City’s trans fat restriction. Ann Intern Med.

[30] NDNS Results from years 1 to 4 combined of the rolling programme for 2008 and 2009 to 2011 and 2012: report in Statics National Diet and Nutrition Survey. https://www.gov.uk/government/statistics/national-diet-and-nutrition-survey-results-from-years-1-to-4-combined-of-the-rolling-programme-for-2008-and-2009-to-2011-and-2012. Accessed 10 Nov 2014.

[31] Downs S (2013). The effectiveness of policies for reducing dietary trans fat: a systematic review of the evidence. Bull World Health Organ.

[32] Barton P, Andronis L, Briggs A, McPherson K, Capewell S (2011). Effectiveness and cost effectiveness of cardiovascular disease prevention in whole populations: modelling study. BMJ.

[33] Turkish Ministry of Health: Healthy Nutrition and Physical Activity Programme. 2013-2017. http://beslenme.gov.tr/content/files/home/turkiye_saglikli_beslenme_ve_hareketli_hayat_programi.pdf. Accsessed 10 Nov 2014.

[34] He FJ, MacGregor GA (2009). A comprehensive review on salt and health and current experience of worldwide salt reduction programmes. J Hum Hypertens.

[35] Bibbins-Domingo K, Chertow GM, Coxson PG, Moran A, Lightwood JM, Pletcher MJ (2010). Projected effect of dietary salt reductions on future cardiovascular disease. N Engl J Med.

[36] Turkish Ministry of Health: Nutrition and Health Survey. 2010. www.sagem.gov.tr/TBSA_Beslenme_Yayini.pdf Accessed 13 Nov 2014.

[37] FAO Country Profiles: Turkey. Food and Agriculture Organisation of the United Nations. http://www.fao.org/countryprofiles/index/en/?iso3=TUR Accessed 16 Nov 2014.

[38] O’Keeffe C, Kabir Z, O’Flaherty M, Walton J, Capewell S, Perry IJ. Modelling the impact of specific food policy options on coronary heart disease and stroke deaths in Ireland. BMJ open. 2013;3(7). doi:10.1136/bmjopen-2013-002837. 10.1136/bmjopen-2013-002837 PMC3703570 23824313

[39] O’Flaherty M (2012). Potential cardiovascular mortality reductions with stricter food policies in the United Kingdom of Great Britain and Northern Ireland. Bull World Health Organ.

[40] Konfino J, Mekonnen TA, Coxson PG, Ferrante D, Bibbins-Domingo K (2013). Projected impact of a sodium consumption reduction initiative in Argentina: an analysis from the CVD policy model--Argentina. PLoS One.

[41] Capewell S, O’Flaherty M (2011). Rapid mortality falls after risk-factor changes in populations. Lancet.

[42] Unal B, Critchley JA, Capewell S (2005). Modelling the decline in coronary heart disease deaths in England and Wales, 1981-2000: comparing contributions from primary prevention and secondary prevention. BMJ.

[43] Briggs AD, Mytton OT, Madden D, O’Shea D, Rayner M, Scarborough P (2013). The potential impact on obesity of a 10 % tax on sugar-sweetened beverages in Ireland, an effect assessment modelling study. BMC Public Health.

[44] National Institute for Health and Care Excellence. Cardiovascular Disease Prevention. 2010. http://www.nice.org.uk/guidance/ph25/resources/cardiovascular-disease-prevention-1996238687173 Accessed 10 Dec 2015.

[45] WHO. Prevention and control of noncommunicable diseases in the European Region:a progress report. 2014.http://www.euro.who.int/en/health-topics/noncommunicable-diseases/ncd-background-information/prevention-and-control-of-noncommunicable-diseases-in-the-european-region-a-progress-report. Accessed 10 Dec 2015.

[46] Ministry of Health of Turkey: National Tobacco Control Programme and Action Plan of Turkey 2008–2012. Ankara; 2008. http://www.tkd-online.org/PDFs/tobacco_plan_en.pdf. Accessed 18 Nov 2015

[47] Ministry of Health of Turkey: Obesity Prevention and Control Program of Turkey (2010–2014). Ankara: Kuban Matbaacilik Yayincilik; 2010. http://www.beslenme.gov.tr/content/files/home/obesity_prevention_and_control_program_of_turkey_2010_2014.pdf. Accessed 16 Nov 2015

[48] Wardlaw JM, Murray V, Berge E, Del Zoppo GJ (2009). Thrombolysis for acute ischaemic stroke. Cochrane Database Sys Rev.

[49] Sandercock PA, Counsell C, Gubitz GJ, Tseng MC (2008). Antiplatelet therapy for acute ischaemic stroke. Cochrane Database Sys Rev.

[50] Stroke Unit Trialists C (2007). Organised inpatient (stroke unit) care for stroke. Cochrane Database Syst Rev.

[51] Algra A, van Gijn J (1999). Cumulative meta-analysis of aspirin efficacy after cerebral ischaemia of arterial origin. J Neurol Neurosurg Psychiatry.

[52] Amarenco P, Labreuche J (2009). Lipid management in the prevention of stroke: review and updated meta-analysis of statins for stroke prevention. Lancet Neurology.

[53] Saxena R, Koudstaal P (2004). Anticoagulants versus antiplatelet therapy for preventing stroke in patients with nonrheumatic atrial fibrillation and a history of stroke or transient ischemic attack. Cochrane Database Systematic Rev.

[54] Zhang H, Thijs L, Staessen JA (2006). Blood pressure lowering for primary and secondary prevention of stroke. Hypertension.

[55] Law M, Wald N, Morris J (2003). Lowering blood pressure to prevent myocardial infarction and stroke: a new preventive strategy. Health Technol Assess.

[56] Ray KK, Seshasai SR, Wijesuriya S, Sivakumaran R, Nethercott S, Preiss D (2009). Effect of intensive control of glucose on cardiovascular outcomes and death in patients with diabetes mellitus: a meta-analysis of randomised controlled trials. Lancet.

[57] Hart RG, Pearce LA, Aguilar MI (2007). Meta-analysis: antithrombotic therapy to prevent stroke in patients who have nonvalvular atrial fibrillation. Ann Intern Med.

[58] Strazzullo P, D’Elia L, Kandala NB, Cappuccio FP (2009). Salt intake, stroke, and cardiovascular disease: meta-analysis of prospective studies. BMJ.

[59] Mozaffarian D, Clarke R (2009). Quantitative effects on cardiovascular risk factors and coronary heart disease risk of replacing partially hydrogenated vegetable oils with other fats and oils. Eur J Clin Nutr.

[60] Jakobsen MU, O’Reilly EJ, Heitmann BL, Pereira MA, Balter K, Fraser GE (2009). Major types of dietary fat and risk of coronary heart disease: a pooled analysis of 11 cohort studies. Am J Clin Nutr.

[61] Dauchet L, Amouyel P, Dallongeville J (2005). Fruit and vegetable consumption and risk of stroke: a meta-analysis of cohort studies. Neurology.

[62] Unal B, Sozmen K, Arik H, Gerceklioglu G, Altun DU, Simsek H (2013). Explaining the decline in coronary heart disease mortality in Turkey between 1995 and 2008. BMC Public Health.

