# Supplementary material for: Estimating the potential contribution of stroke treatments and preventative policies to reduce the stroke and ischemic heart disease mortality in Turkey up to 2032: a modelling study
Source: BMC Public Health. 2016 Jan 19;16:46. doi: 10.1186/s12889-015-2655-8 (PMC4717543; doi:10.1186/s12889-015-2655-8)
Supplement: Additional file 1: — Technical Appendix. (DOC 417 kb) [file 12889_2015_2655_MOESM1_ESM.doc]

**TECHNICAL APPENDICES**

**Title:** Estimating the potential contribution of stroke treatments and preventative policies to reduce the stroke and ischemic heart disease mortality in Turkey up to 2032: A modelling study

**Authors:** Duygu Islek, Kaan Sozmen, Belgin Unal, Maria Guzman-Castillo, Ilonca Vaartjes, Julia Critchley , Simon Capewell, Martin O’Flaherty

| **CONTENTS**  ***List of abbreviations***  **1. Supplemental Methods**  *1.1. Overview of the Turkish Stroke Model*  *1.2. Data Sources*  *1.2.1. Population Data*  *1.2.2. Data for Prevalence of Stroke*  *1.2.3.Data for Incidence of Stroke*  *1.2.4. Data for Mortality and Case-Fatality Rates*  *1.2.5. Data for Calculation of Transition Probabilities*  *1.2.6. Data for Medical Therapies*  *1.2.7. Data for Population Level Policy Scenarios* |
| --- |
| *1.3. Baseline Scenario*  *1.4. Modelling Medical Therapies in Turkish Stroke Model*  *1.5. Calculation of DPPs*  **2. Supplemental Tables**  **3. Supplemental Figure and Figure Legends**  ***References*** |

| DPPs | Deaths Prevented or Postponed |
| --- | --- |
| CVD | Cardiovascular Disease |
| TurkStat | Turkish Statistical Institute |
| MoH | Ministry of Health |
| TP | Transition Probability |
| BP | Blood Pressure |
| HbA1c | Hemoglobin A1c |
| AF | Atrial Fibrillation |
| PUFA | Polyunsaturated fat |
| RRR | Relative Risk Reduction |

***List of abbreviations***

1. **SUPPLEMENTAL METHODS**
   1. **Overview of the Turkish Stroke Model**

The Ischemic Stroke Model is a cell-based model, constructed using Microsoft Excel, which integrates available country-specific epidemiological data to estimate the number of deaths prevented or postponed (DPPs) with the impact of medical therapies and population policies. The model calculates the DPPs for each medical therapy and each population based policy scenario separately and requires sex specific data for 10 year age bands (35 to 94 years of age). The tables included in this supplementary document provide details about the data sources, estimates, assumptions and calculations used in the model.

- 1. **Data Sources**
     1. **Population data**

We obtained the population data for year 2012 from Turkish Statistical Institute (TurkStat).

- - 1. **Data for Prevalence of Stroke**

We obtained prevalence of ischemic stroke from a nationwide study conducted by Ministry of Health in 2012. Using the population and ischemic stroke prevalence data from Chronic Diseases and Risk Factor Study of Turkish Ministry of Health (MoH) we calculated the number of ischemic stroke patients in total population.

- - 1. **Data for Incidence of stroke**

We modelled the incidence of ischemic stroke in Turkey for the stroke-free population. Since there was no data available for incidence of stroke in Turkey, we used DISMOD II Software to make an estimation. DISMOD II calculates the incidence by using population data, total mortality rate, prevalence of stroke, remission rate (assumed to be zero) and mortality of major stroke. In order to inform DISMOD estimation, we added as an input incidence from an Iranian stroke cohort study and found an estimate for Turkey that is not only consistent with Turkish parameters, but also with a comparable regional population, similar in terms of Non Communicable Diseases (NCD) epidemiology.

- - 1. **Data for Mortality and Case-Fatality Rates**

We obtained number of deaths for age groups 35 to 94 from TurkStat for the year 2012. The model also required total Cardiovascular Diseases (CVD) deaths data with the ICD 10 code from 100 to 199 which we also obtained from TurkStat database for year 2012. The case fatality rates of ischemic stroke (both minor and major) were calculated by analyzing the hospital based Aegean Stroke Registry database with Kaplan-Meier Method. Aegean Stroke Registry was used with the collaboration of researchers of Egean University Hospital . All the input data were specified for age and sex.

- - 1. **Data for Calculation of Transition Probabilities**

The ischemic stroke model was a Markov model consisting finite number of health states reflecting the natural history of ischemic stroke. We assumed that a close cohort of individuals is free of stroke at the start of the simulation and every year the individuals can move from one health state to another. These movements between states are governed by probabilities called Transition probabilities (TPs). In total, the model calculates 16 TPs to estimate DPPs for each medical therapy or policy intervention scenario. For definition and calculations of TPs, see Table I.

- - 1. **Data for Medical Therapies**

The model required data on uptake and clinical efficacy for acute care and primary and secondary prevention of ischemic stroke.

**Clinical Efficacy of Interventions**

The model calculates the clinical efficacy of interventions using relative risk reductions (RRRs) obtained from meta-analyses, and randomised clinical trials. For the definition and source paper of the RRRs see Table II.

**Uptake of medical treatments:**

1. **Acute Stroke Treatment:** The model required data of the % of the eligible ischemic stroke patients that actually receive thrombolysis, aspirin treatment and the % of the eligible population that are treated in a stroke unit. See Table III for definition, main data source, present uptake value for acute stroke treatment.
2. **Secondary Prevention Treatment:** Model required data of the % of the ischemic stroke patients that actually receive aspirin, statin, warfarin and antihypertensive medical treatment and % of ischemic stroke patients that have actually quit smoking. See Table IV for definition, main data source, present uptake value for secondary prevention treatment.
3. **Primary Prevention Treatment:** Model required data of the % of general population that has controlled blood pressure (BP), controlled Hemoglobin A1c (HbA1c) value, % of general population that quit smoking, % of the population with Atrial Fibrillation (AF) that receive warfarin treatment. See Table V for definition, main data source, present uptake value for primary prevention treatment.
   - 1. **Data for Population Level Policy Scenarios**

We simulated population level scenarios for desired reduction in salt consumption in grams per day, for desired reduction in trans-fat and saturated fat in % of energy per day, for increase in fruit and vegetable intake in units per day. For reduction in smoking prevalence in the population we simulated a decrease in prevalence in 10 and 20 years. For these population level scenarios model required a base estimate of risk reduction in CVD deaths and an assumption for the age effect. We assumed the same age effect for the scenarios previously observed with cholesterol based on a meta-analysis of individual data on blood cholesterol and vascular mortality. See Table VI for the base estimates of risk reduction used for each population level policy scenario.

- 1. **Baseline Scenario**

In the baseline scenario we assumed no change will happen during 10 and 20 years in the present uptake rates of medical therapies or population level uptakes of nutrients and population smoking prevalence.

- 1. **Modelling Medical Therapies in Turkish Stroke Model**

The model required data of medical therapies at three levels: acute stroke treatment (uptake rates of thrombolysis, aspirin, stroke unit), secondary prevention (uptake rates of aspirin, statin, warfarin, antihypertensives and % of people who actually quit smoking), primary prevention (rates of eligible population who have blood pressure control, HBA1c Control, Smoking Cessation rate in the population and present uptake of warfarin treatment).

For uptake rates of these medical therapies, we simulated three scenarios:

(a) **an optimal scenario**; simulating an increase in the uptake levels that are already reached in more developed countries, as an exemplar country, the Netherlands.

(b) **a conservative scenario**; assuming a modest 10% relative increase in the current uptake levels.

(c) **a feasible scenario**; an intermediate point, more realistic change halfway between the current situation and the optimistic scenario.

For secondary prevention, the uptake levels of statin, warfarin and smoking cessation was already higher than the present uptake level in the exampler country, the Netherlands, so we assumed 10% relative increase for three scenarios. We also simulated 10% relative increase for blood pressure and HbA1c control. The simulated scenarios for the uptake rates of medical therapies can be seen in Table VII.

- 1. **Calculation of DPPs**

The primary output of the model is the DPPs. The model applies the relative risk reduction quantified in previous randomised controlled trials and meta-analyses to estimate the reduction in CVD deaths.

An example of the calculation method used for estimating the DPPs with a population level policy scenario is provided below.

***Calculation example of DPPs***

- Intervention: 3 gr change in salt consumption
- Age-gender group: 65 -74 male
- Number of healthy individuals: 699,938

5 gr salt reduction has an effect of 0.17 on cardiovascular disease and deaths. In order to calculate the impact of 3 gr reduction in salt consumption we do the following correction (0.17*3/5=0.102). We take into account the age effects for each intervention risk reduction value, hence for age group 65-74 age effect is 0.53 and this number is multiplied with 0.102 to get the age effect adjusted risk reduction value (0.053631). Hence, effectiveness value is 1-0.053631=0.94637. Salt reduction shows effect by changing the following transition probabilities, as result effectiveness value has to be multiplied with these TPs TP1;Population free stroke to first minor stroke (1st year), TP2; Population free stroke to first major stroke, TP3; Population free stroke to CVD deaths and TP4; Population free stroke to non CVD deaths.

If we demonstrate it in more detail for moving from cycle 18 to cycle 19 for men aged 65-74. (See Figure I)

1. **TP1: Population free stroke to first minor stroke (1st year)**

Number of healthy people (699938)* TP 1 (0.0078000)* Effectiveness SALT (0.94637) =5167

1. **TP2: Population free stroke to first major stroke**

Number of healthy people (699938) * TP 2 (0.0022000)* Effectiveness SALT (0.94637) + Number of people with minor stroke in 1st year (5401)* TP 6 (0.0124224) + Minor stroke subsequent year (90578) *TP 10 (0.0400000) + Major stroke (62745) * TP 16 (0.9647799) =65730

1. **CVD Death total:**

Number of deaths previous year (141435) + (Number of healthy people (699938) *TP 3 (0.0064749)* Effectiveness SALT (0.94637)+ Number of people with minor stroke in 1st year(5401)* TP 7 (0.0108679) +Minor stroke subsequent year (90578) *TP 11 (0.0056223) = 147884

1. **Non CVD Deaths:**

Non CVD deaths previous year (555909) + Number of healthy people (699938)* TP4 (0.0293991)* Effectiveness SALT (0.94637)+(Number of people with minor stroke-1st year (5401)* TP 8 (0.0042264) + Minor stroke subsequent year (90578)* TP 12 (0.0014056)+ Major stroke (62795)*TP 15 (0.0098616) = 576152

1. **Number of Healthy People:**

Number of healthy people (699938)* (1-(Effectiveness SALT (0.94637)* TP1 (0.0078000)+Effectiveness SALT (0.94637)* TP 2 (0.0022000)+ Effectiveness SALT (0.94637)*TP 3 (0.0064749) + Effectiveness SALT (0.94637)*TP 4 (0.0293991) =669551

Number of CVD deaths prevented is 5,505 for stage 19.

See Table VIII for the comparison of number of people for cycle 19 stratified with health states. A similar approach was used to estimate the effect of increasing the uptake of medical treatments.

1. **SUPPLEMENTAL TABLES**

**Table I. Transition Probabilities data sources used in the stroke model**

|  | **Definition** | **Main Data Source** | **Calculation** | **Comments** |
| --- | --- | --- | --- | --- |
| Stroke Free Population | The total stroke free population in the year 0. | Address-based Population Registry System, Turkish Statistical Institute, 2012 , DISMOD II | Total Population  – Ischemic Stroke Patients | Ischemic Stroke Patients were calculated as ‘Total Population*Incidence of Ischemic Stroke in 2012’ |
| TP1:Population free of Stroke to first Minor Stroke (1st year) | The probability for the stroke free population to have first minor stroke in the year 1. | DISMOD II , Egean Stroke Registry , TurkSTAT 2012 | (Ischemic Stroke Patients*Proportion of Minor Stroke)/Total Population | Proportion of minor strokes were calculated from Egean Stroke Registry. The main limitation was the small numbers of minor stroke admissions which might have caused a selection bias. |
| TP2: Population free of Stroke to first Major Stroke | The probability for the stroke free population to have first major stroke in the year 1. | DISMOD II , Egean Stroke Registry TurkSTAT 2012 | (Ischemic Stroke Patients*(1-Proportion of Minor Stroke))/Total Population | Proportion of minor strokes were calculated from Egean Stroke Registry. 275 of 290 admissions were major stroke which might have caused a selection bias and an over estimation. |
| TP3: Population free of Stroke to CVD Deaths | The probability for the stroke free population to die from CVD causes in the year 1. | Turkish Statistical Institute 2012 | (Proportion of CVD deaths*Total deaths)/Total Population | Total deaths and the proportion of CVD deaths were estimated for 2013 using the observed data in 2012. |
| TP4:Population free of Stroke to NonCVD Deaths | The probability for the stroke free population to die from Non CVD causes in the year 1. | Turkish Statistical Institute 2012 | ((1-Proportion of CVD deaths)*Total deaths)/Total Population | Total deaths and the proportion of CVD deaths were estimated for 2013 using the observed data in 2012. |
| TP5:Population free stroke to population free stroke | - | - | 1-(TP1+TP2+TP3) | - |
| TP6: First minor stroke (1st year) to first major stroke | The probability of recurrent stroke in ischemic stroke patients after 1 year | Based on expert opinion and Literature |  |  |
| TP7: First Minor Stroke (1st year) to CVD Deaths | The probability for the minor stroke patients to die from CVD causes in the year 1. | Based on estimation using Dutch Data  (% of deaths due to CVD after 1 year in patients that were hospitalised for a first ischemic stroke in 2000 in the Netherlands) | - | The death numbers among minor ischemic stroke patients were very small in Egean Stroke Registry, we therefore used the death ratio among stroke patients in Dutch data in order to estimate the death numbers in Turkish ischemic stroke patients. |
| TP8: First Minor Stroke (1st year) to NonCVD Deaths | The probability for the minor stroke patients to die from NonCVD causes in the year 1. | Based on estimation using Dutch Data  ( % of deaths due to CVD after 1 year in patients that were hospitalised for a first ischemic stroke in 2000 in the Netherlands.) | - | The death numbers among minor ischemic stroke patients were very small in Egean Stroke Registry, we therefore used the death ratio among stroke patients in Dutch data in order to estimate the death numbers in Turkish ischemic stroke patients. |
| TP9: First minor stroke (1st year) to first minor stroke subsequent years |  |  | 1-(TP6+TP7+TP8) |  |
| TP10: First Minor Stroke in Subsequent Years to First Major Stroke | % of recurrent stroke in ischemic stroke patients after 5 year | Based on expert opinion |  |  |
| TP11: First Minor Stroke in Subsequent Years to CVD Deaths | The probability for the minor stroke patients to die from CVD causes 1 year after first admission | Dutch Data (All deaths after 5 year in patients that were hospitalised for a first ischemic stroke in 2000 in the Netherlands.) |  | The initial year of the Turkish cohort is 2012, we therefore estimated the deaths in 5 years using the Dutch data. |
| TP12:First Minor Stroke in Subsequent Years to NonCVD Deaths | The probability for the minor stroke patients to die from nonCVD causes 1 year after first admission. | Dutch Data (All deaths after 5 year in patients that were hospitalised for a first ischemic stroke in 2000 in the Netherlands.) |  | The initial year of the turkish cohort is 2012, we therefore estimated the deaths in 5 years using the Dutch data. |
| TP 13:First minor stroke subsequents years to first minor stroke subsequent years |  |  | 1-(TP10+TP11+TP12) |  |
| TP14: First Major Stroke to CVD Deaths | The probability for the major stroke patients to die from CVD causes | Dutch Data(All deaths after 1 year in patients that were hospitalised for a first ischemic stroke in 2000 in the Netherlands.) |  | The death numbers among major ischemic stroke patients were very small in Egean Stroke Registry, we therefore used the death ratio among stroke patients in Dutch data in order to estimate the death numbers in Turkish ischemic stroke patients. |
| TP15: First Major Stroke to NonCVD Deaths | The probability for the major stroke patients to die from nonCVD causes | Dutch Data (All deaths after 1 year in patients that were hospitalised for a first ischemic stroke in 2000 in the Netherlands.) |  | The death numbers among major ischemic stroke patients were very small in Egean Stroke Registry, we therefore used the death ratio among stroke patients in Dutch data in order to estimate the death numbers in Turkish ischemic stroke patients. |
| TP 16:First major stroke to first major stroke |  |  | 1- (TP14+TP15) |  |

**Table II.** Clinical efficacy of interventions: relative risk reductions obtained from meta-analyses, and randomised clinical trials

| Treatments | Relative risk reduction | Definition | Source paper |
| --- | --- | --- | --- |
| Acute Stroke Treatment | | | |
| Thrombolysis | 11% (95% CI: 5-16) | RRR for death or dependency if (tPA) within 4.5 hours | Wardlaw JM (2009) |
| Aspirin | 2,6% (95% CI: 0.4-4) | RRR for death or dependency if treatment is 160-300mg once daily, started within 48h of onset | Sandercock (2008) |
| Stroke Unit | 6,1%  (95% CI:0,0009-11) | RRR for death or dependency. Broad definition for stroke unit | Stroke Unit Trialist Collaboration (2007). |
| Secondary Prevention | | | |
| Aspirin | 3% (95% CI: 6-19) | RRR for vascular events (vascular death, stroke or if treatment is Aspirin at any dose above 30 mg daily. | Algra A. (1999) |
| Statin | 12% (95% CI: (-1)-21) | RRR for recurrent stroke if LDL reduces by 1 mmol/L. | Amarenco P (2009) |
| Warfarin | 61% (95% CI: 37-75) | RRR for recurrent stroke or systemic embolism among stroke patients with Transient Ischemic Attack or minor stroke due to atrial fibrillation when treated with anticougalation with adjusted-dose warfarin (international normalised ratio of 2.0-3.0) | Saxena R (2004) |
| BP Control | 34% (95% CI: 21-44) | RRR based on BP reduction 4-25 mmHG systolic or 3-13 Hg diastolic. Dose daily one standard dose or 3 half standard dose RRR for recurrent stroke | Zhang H (2006) |
| Smoking Cessation | 48% (95% CI: 29-57) | RRR for death or dependency | Hankey GJ (2010) |
| Primary Prevention | | | |
| BP Control | 46% (95% CI: 35-55) | RRR based on BP reduction 5 mmHg. This reduces the risk of stroke by an estimated 34% and ischemic heart disease by 21% from any pre-treatment level | Law M. (2003) |
| HbA1C Control | 7% (95% CI: 4-19) | RRR based on 0,9% HbA1C reduction. | Ray KK (2003) |
| Statin | 36% (95% CI: 22-48) | RRR based on 1mmol/L reduction/ RRR for recurrent stroke | Amarenco P (2009) |
| Smoking Cessation | 48% (95% CI: 29-57) | RRR for death or dependency | Hankey GJ (2010) |
| Warfarin | 64% (95% CI: 49-74) | RRR based on a meta-analysis with twenty-nine trials included compared with the control, adjusted-dose warfarin (6 trials) and antiplatelet agents (8 trials) reduced stroke by 64% | Hart RG et al (2007) |

**Table III.** Data sources and present uptakes for acute stroke treatment

| Akut Stroke Treatment | Definition | Main Data Source | Present Uptake | Comment |
| --- | --- | --- | --- | --- |
| Thrombolysis | % of ischemic stroke patients that have recieved TPA treatment started within 4,5 h after onset of symptoms of ischemic stroke | Literature and expert opinion | 0,01 | We have the data of two hospital-based studies which we assumed can not be generalised for the whole country. According to avoid selection bias, we used the literature and expert opinion. |
| Aspirin | % of ischemic stroke patients that have recieved aspirin treatment 160-300mg once daily | Egean Stroke Registry | 0,50 | We used the data base of a hospital-based stroke registry. |
| Stroke Unit | % of the eligible population that actually are treated at a stroke unit | Expert Opinion | 0,00 | There is no data for the stroke unit facilities considering whole Turkey. |

**Table IV.** Data sources and present uptakes for secondary prevention treatment of stroke

| Secondary Preventıon | Definition | Main Data Source | Present Uptake |
| --- | --- | --- | --- |
| Aspirin | the % of the eligible population that actually recieved aspirin treatment at any dose above 30 mg daily | Balçova Heart Study | 0,26 |
| Statin | the % of the eligible population that actually recieve the treatment | Chronic Diseases and Risk Factor Study of MoH | 0,48 |
| Warfarin | % of ischemic stroke patients that have recieved warfarin treatment | Balcova Heart Study and  DEU Stroke and Cost Study database | 0,14 |
| Anti HT Uptake Rate | % of the eligible population that actually recieve the treatment | WHO Study on prevention of recurrences of Stroke (WHO-Premise) | 0,35 |
| Smoking Cessation | % of ischemic stroke patients that have quit smoking | Balcova Heart Study | 0,30 |

**Table V.** Data sources and present uptakes for primary prevention treatment of stroke

| Primary Preventıon Treatment | Definition | Main Data Source | Present Uptake |
| --- | --- | --- | --- |
| BP Control | the % of the eligible population that recieved prescription for BP control | Chronic Diseases and Risk Factor Study of MoH | 0,07 |
| HBA1C Control | the % of the eligible population that recieved prescription for HBA1C control | Turkish Diabetes Epidemiology Study (TURDEPII) | 0,49 |
| Smoking Cessation | % of general population that quit smoking | TurkStat, Global Adult Tobacco Survey, 2012 | 0,04 |
| Warfarin | the % of the eligible population that actually recieved warfarin treatment | Egean Stroke Registry database | 0,30 |

**Table VI.** Definitions and source paper of base estimates in risk reduction used for population level scenarios

| Policy | Definition of Base Estimate | Base Estimate of Risk Reduction | Source Paper |
| --- | --- | --- | --- |
| Salt | Risk reduction of stroke by 5gr change in daily salt intake | 0,17 | Strazzullo et al. (2009) |
| Transfat | Risk reduction of Coronary Heart Disease by replacing 1% of energy from trans-fat with unsaturated fats. We assumed for stroke half the effect. | 0,05 | Mozaffarian D. (2009) |
| Saturated Fat | Risk reduction of Coronary Heart Disease by replacing 5% of energy from saturated fat with Polyunsaturated fats (PUFAs). We assumed that effect size on stroke is 0.5 compared to Coronary Heart Disease. | 0,13 | Jakobsen (2009). |
| Fruit and Vegetables | Risk reduction of stroke on change in 1 unit of fruit and vegetables. | 0,05 | Dauchet (2005) |
| Smoking | Beta coefficient for a change in 1% prevalence in smoking. | 0,30 | Unal(2013) |

**Table VIII.** Comparison of number of people for example of calculations in model (cycle 19) stratified with health states.

|  | Baseline scenario | Salt reduction | Difference |
| --- | --- | --- | --- |
| TP1:Population free stroke to first minor stroke (1st year) | 5,212 | 5,167 | 45 |
| TP2 Population free stroke to first major stroke: | 68,234 | 65,730 | 2,504 |
| CVD Death total | 153,389 | 147,884 | 5,505 |
| Non CVD Deaths | 596,992 | 576,152 | 20,840 |
| Healthy | 637,579 | 669,551 | -31,972 |
| Total number of people | 1,556,055 | 1,556,055 | 0 |

**Figure I:** Transition Probabilities between health states in Turkish Stroke Model


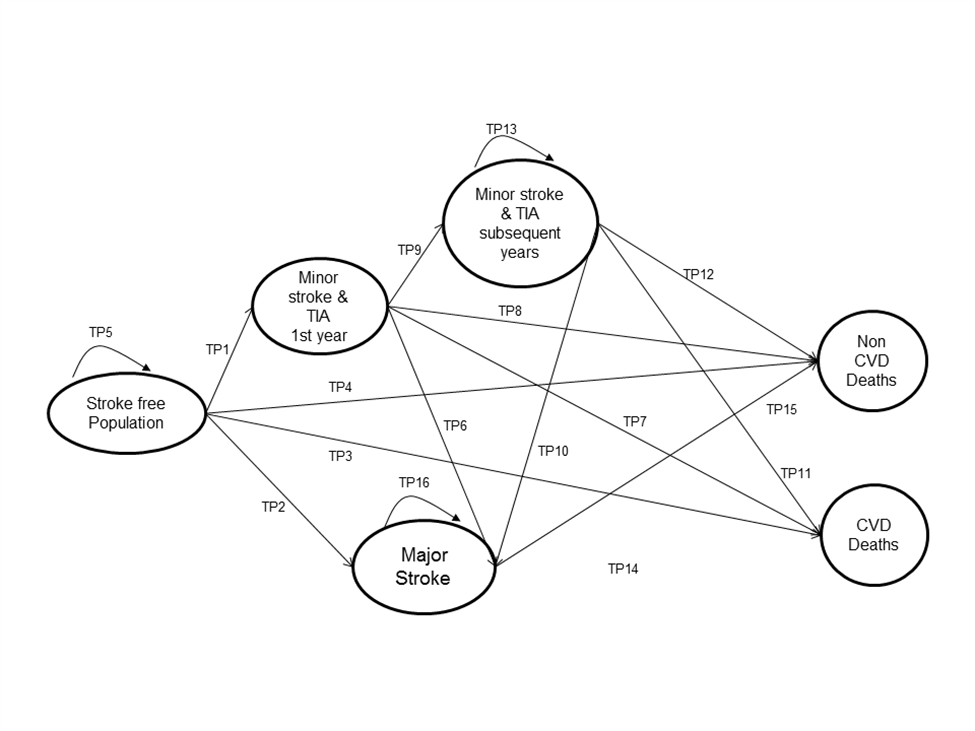


References
